# King’s Health Partners’ Prostate Cancer Biobank (KHP PCaBB)

**DOI:** 10.1186/s12885-017-3773-8

**Published:** 2017-11-22

**Authors:** S. R. Saifuddin, W. Devlies, A. Santaolalla, F. Cahill, G. George, D. Enting, S. Rudman, P. Cathcart, B. Challacombe, P. Dasgupta, C. Galustian, A. Chandra, S. Chowdhury, C. Gillett, M. Van Hemelrijck

**Affiliations:** 1 School of Cancer and Pharmaceutical Sciences, Translational Oncology & Urology Research, King’s College London, London, UK; 2 Faculty of Medicine, KU Leuven, Leuven, Belgium; 3 Oncology Department, Guy’s and St Thomas’ NHS Foundation Trust, London, UK; 4 Urology Centre, Guy’s and St Thomas’ NHS Foundation Trust, London, UK; 5 MRC Centre for Transplantation, Faculty of Life Sciences & Medicine, King’s College London, London, UK; 6 Pathology Department, Guy’s and St Thomas’ NHS Foundation Trust, London, UK; 7 King’s Health Partners Cancer Biobank, Guy’s Hospital, London, UK

**Keywords:** Prostate cancer, Biobank, Data resource profile, Clinical database, Tissue repository

## Abstract

The KHP PCaBB was established in 2013 and recruits donors from the Urology or Oncology Departments at Guy’s Hospital in London (UK). Prostate cancer patients may be approached to give their consent for biobanking at any point in their treatment pathway, which allows residual material from their earlier diagnosis to be transferred and used by the Biobank. Currently, patients are specifically asked to donate samples of blood and surplus prostate tissue as well as permitting access to their clinical and pathological data that continues to be added throughout the course of their disease. Between 2013 and 2015, 549 prostate cancer patients gave their consent to the biobank and, the tissue repository collected 489 blood samples, 120 frozen prostate tissue samples and 1064 formalin fixed paraffin embedded diagnostic blocks.

Prostate cancer has become a chronic disease in a large proportion of men, with many men receiving multiple subsequent treatments, and their treatment trajectory often spanning over decades. Therefore, this resource aims to provide an ideal research platform to explore potential variations in treatment response as well as disease markers in the different risk categories for prostate cancer.

A recent audit of the KHP PCaBB revealed that between 2013 and 2015, 1796 patients were diagnosed with prostate cancer at King’s Health Partners (KHP), out of which 549 (30.6%) gave their consent to KHP PCaBB. Comparisons between demographic and clinical characteristics of patients who had consented compared to the total patient population revealed that the KHP PCaBB is demographically representative of the total prostate cancer patient population seen in Guy’s and St Thomas’ NHS Foundation Trust (GSTT). We observed no differences in distribution of ethnicity (*p* = 0.507) and socioeconomic status (*p* = 0.097). Some differences were observed in clinical characteristics, specifically with treatment type – which differed significantly between the patients who had given consent and total patient population.

The KHP PCaBB has thereby amassed a rich data and tissue repository that is largely reflective of both the demographic and clinical diversity within the total prostate cancer patient population seen at KHP, making it an ideal platform for prostate cancer research.

## Background

Prostate cancer affects approximately 1.1 million men worldwide [1], yet much remains unknown about this complex disease. The heterogeneous nature of prostate cancer makes it difficult to elucidate the specific mechanisms of disease aetiology and progression. As such, urological biobanking aims to provide a resource to bridge this gap through the translation of research into practical applications, thereby spurring advancements in both public health and personal patient care [2].

Currently, more than 12 prostate biorepositories in the UK have been established for current or prospective research such as ProtecT, ProMPT and the Wales Cancer Bank [3]. While all biobanks record clinical information of disease, few include the demographic characteristics of donors; leaving pockets of unexplored territory in prostate cancer research.

**Fig. 1:**
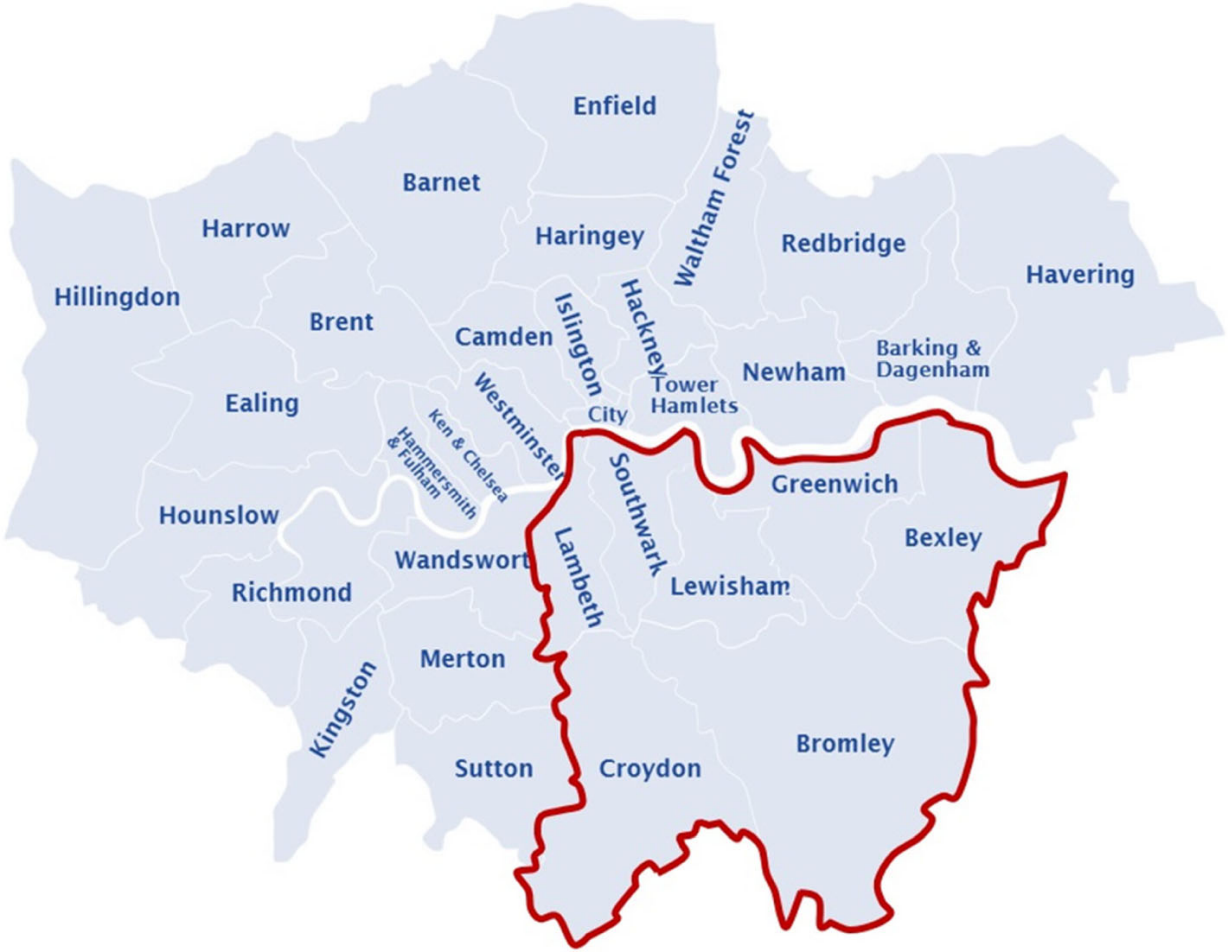
Catchment area of KHP (highlighted in red). Image adapted with permission from London City Council Government Directory [20]

**Fig. 2:**
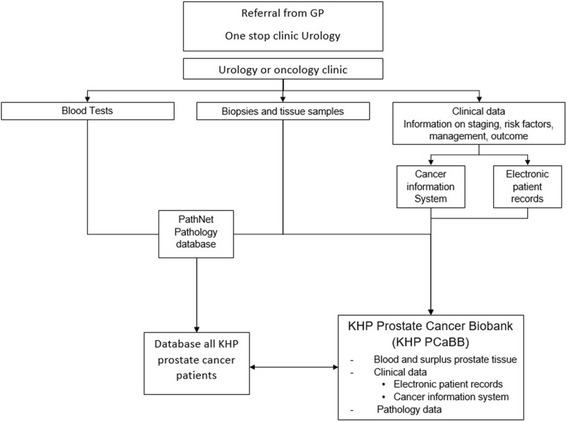
Treatment pathway of prostate cancer patients seen at KHP

King’s Health Partners (KHP) sees nearly 20,000 suspected cancer patients each year, drawn from a referral base of over two million people across South East London (Fig. 1) and treats approximately 6500 cancer patients [4]. Because of its outreach, patients referred to KHP have a wide ethnic and socioeconomic diversity, hence its biobanks are a valuable and often unique resource for medical research of various diseases, including prostate cancer. The KHP Prostate Cancer Biobank (KHP PCaBB) was established in 2013 as part of the KHP Cancer Biobank and recruit donors from the Urology or Oncology Departments at Guy’s Hospital. Patients may be approached to give their consent for biobanking at any point in their treatment pathway (Fig. 2), which allows residual material from their earlier diagnosis to be transferred and used by the Biobank. As such, the KHP PCaBB consists of patients who gave consent for biobanking after 1 January 2013, but whose samples may have been collected before that date. Prostate cancer patients are currently asked to donate samples of blood and surplus prostate tissue as well as permitting access to their data. Clinical data continues to be added throughout the course of their disease.

A recent audit of the KHP Prostate Cancer Biobank revealed that between 2013 to 2015, 1796 patients were diagnosed with prostate cancer at KHP and 549 (30.6%) gave their consent to KHP PCaBB. Comparisons between demographic and clinical characteristics of patients who had given consent compared to the total patient population revealed that the KHP PCaBB is demographically representative of the total prostate cancer patient population seen in Guy’s and St Thomas’ NHS Foundation Trust (GSTT). We observed no differences in distribution of ethnicity and socioeconomic status (Table 1). Greater differences were observed in clinical characteristics, with treatment type differing significantly between the patients who had given consent and total patient population (Table 2).

**Table 1:** Demographic information of KHP PCaBB participants from Jan 2013-Dec 2015

Demographic Variable	Consented (*n* = 549)
Age at diagnosis (years)	
< 50	42 (7.7)
50–59	160 (29.1)
60–69	262 (47.7)
70–79	76 (13.8)
80–89	8 (1.5)
> 90	0 (0.0)
Unrecorded	1 (0.2)
Ethnicity	
White	246 (44.8)
Black	87 (15.8)
Asian	11 (2.0)
Mixed	5 (0.9)
Other	4 (0.7)
Unrecorded	196 (35.7)
Socioeconomic background	
Low	462 (84.2)
Middle	21 (3.8)
High	54 (9.8)
Missing	12 (2.2)

**Table 2:** Clinical information of KHP PCaBB participants from Jan 2013-Dec 2015

Clinical Variable	Consented (n = 549)
Treatment type	
Surgery	357 (65.0)
Active Monitoring	74 (13.5)
Anti-cancer drug regimen (Hormone Therapy)	98 (17.9)
Anti-cancer drug regimen (Cytotoxic Chemotherapy)	2 (0.4)
Brachytherapy	18 (3.3)
Specialist palliative care	0 (0.0)
Mean PSA (SD) at diagnosis (μg/L)	42.77 (±374.927)
Gleason Score	
6	74 (13.5)
7	358 (65.2)
8	44 (8.0)
9	53 (9.7)
10	3 (0.5)
Unrecorded	19 (3.5)
Gleason Group	
Grade 1	74 (13.5)
Grade 2	247 (45.0)
Grade 3	111 (20.2)
Grade 4	44 (8.0)
Grade 5	56 (10.2)
T stage	
TX	42 (7.7)
T0	28 (5.1)
T1	4 (0.7)
T1a	2 (0.4)
T1b	0 (0.0)
T1c	16 (2.9)
T2	106 (19.3)
T2a	153 (27.9)
T2b	15 (2.7)
T2c	60 (10.9)
T3	17 (3.1)
T3a	69 (12.6)
T3b	34 (6.2)
T4	3 (0.5)
T4a	0 (0.0)
N stage	
NX	191 (34.8)
N0	334 (60.8)
N1	24 (4.4)
N2	0 (0.0)
M stage	
MX	221 (40.3)
M0	315 (57.4)
M1	5 (0.9)
M1a	1 (0.2)
M1b	5 (0.9)
M1c	2 (0.4)
Risk Category	
Localised prostate cancer	
Low risk	83 (15.1)
Intermediate risk	252 (45.9)
High Risk	157 (28.6)
Regionally metastatic/ Locally advanced	23 (4.2)
Distant metastasis	25 (4.6)
Unrecorded	9 (1.6)
Comorbidities	
Mean number of comorbidities (SD)	1.9 (±2.021)
Previous/other cancer	20 (3.6)
Cardiovascular disease	218 (39.7)
HIV or Infectious Diseases	5 (0.9)
Hyperlipidaemia and hypercholesterolemia	87 (4.8)
Diabetes	68 (3.8)
Psychological	32 (5.8)
Mean number of medications (SD)	2.213 (±2.529)
Erectile dysfunction medication	216 (39.3)
Outcome	
Disease stable	444 (80.9)
Discharged to GP	56 (10.2)
Treatment discharged to GP	17 (3.1)
Discharged to different hospital	4 (0.7)
Progression	7 (1.3)
Progression to metastasis	3 (0.5)
Recurrence	2 (0.4)
Death	6 (1.1)
Awaiting treatment	2 (0.4)
Refused further treatment	1 (0.2)
Treated privately	1 (0.2)
Lost to follow up	6 (1.1)
Involvement in clinical trial	34 (6.2)

The KHP PCaBB is both a clinically and demographically representative database; therefore, this resource aims to provide an ideal research platform to explore potential variations in treatment response as well as disease markers in the different risk categories for prostate cancer.

**Fig. 3:**
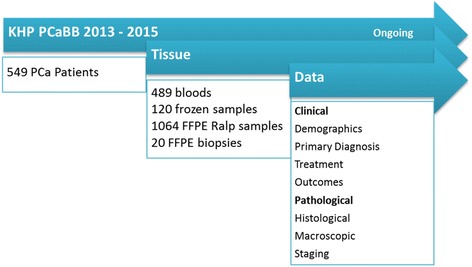
Schematic description of the data and tissue available in King’s Health Partners Prostate Cancer Biobank (KHP PCaBB)

All cohort descriptions presented below are based on the 549 patients who gave consent between January 2013 and December 2015 (Tables 1 and 2). Recruitment to the Biobank is ongoing (Fig. 3).

## Construction and content

### Data repository

The KHP Cancer Biobank is licenced by the Human Tissue Authority and is a National Health Service Research Ethics Committee [5] approved Tissue Bank with generic ethical approval to supply bioresources. All clinical and demographic patient data was obtained using routinely collected information from the Electronic Patient Records and Cancer Information System at GSTT (Table 3).

**Table 3:** Overview of the demographic and clinical dataset

Basics	Sample Size	1796
	Patients Consented	549
	Number of variables	13
	Involvement in clinical trial	Yes /No
Demographics	Demographics	Age at diagnosis Ethnicity Postcode
Prostate Cancer Characteristics	Diagnostic markers	PSA Gleason score TNM stage
Treatment	Treatment type	Surgery, Active monitoring, Anti-cancer drug regimen (hormone therapy), Anti-cancer drug regimen (cytotoxic chemotherapy), Brachytherapy, Specialist palliative care
	Comorbidities	All, Previous/other cancers, Cardiovascular disease, HIV or infectious disease, Hyperlipidaemia and hypercholesterolemia, Diabetes, Psychological
	Medication	Total number of medications, Erectile dysfunction medication
	Outcome	Disease stable, Discharged to GP, Treatment discharged to GP, Discharged to different hospital, Progression, Progression to metastasis, Recurrence, Death, Awaiting treatment, Refused further treatment, Treated privately, Lost to follow up

The following abbreviations have been used: Prostate Specific Antigen (PSA), Human Immuno-Deficiency Virus (HIV) and General Practitioner (GP)

### Tissue repository

The KHP PCaBB tissue repository has collected 489 blood samples, 120 frozen prostate tissue samples and 1064 formalin fixed paraffin embedded (FFPE) diagnostic blocks which are obtained via radical prostatectomy, as well as 20 cores taken as additional research samples during biopsies (Fig. 3).

Donated prostate tissue is collected when it is surplus to diagnostic purposes. Surgical specimens are collected fresh from the operating theatre (i.e. biopsies or radical prostatectomies) and immediately dissected by the prostate histopathologists. They identify macroscopic areas of tumour which are then sampled, snap frozen and safely stored at −80°C. The remaining tissue is fixed in formalin for subsequent paraffin embedding and diagnosis. After six months, residual FFPE tumour blocks are transferred to KHP PCaBB for research use.

Pathological information from the diagnostic specimen is recorded in the KHP PCaBB database and includes weight, lymph node dissection (if any), histological subtype, Gleason Score, presence of positive surgical margins, pathological TNM stage, location and tumour distribution within the prostate, dimension of largest tumour nodule, spread and involvement of disease within the prostate as well as presence of extra-prostatic spread to seminal vesicles or bladder neck. For prostate tissue obtained via biopsy, pathological information stored includes the number of cores taken, site of cores obtained (base, md-zone or apex; left or right), type of biopsy (trans-rectal ultrasound (TRUS) / transperineal (TP) / transurethral resection of prostate (TURP)), TRUS volume, type of carcinoma, number of positive cores, Gleason Score, pathological TNM stage, maximum cancer length, site of maximum cancer length and presence of perineal invasion.

From the whole blood samples a number of derivatives are created including, peripheral blood mononuclear cells (PBMC), plasma, serum and red blood cells. The derivatives are aliquoted, frozen and safely stored at −80°C.

To ensure comprehensive data profiles for each patient, pathological data are linked to clinical patient data using the patient’s unique hospital identification number.

A more detailed overview of the demographic and clinical data recorded is given below.

### Demographics

Demographic patient information includes age at diagnosis, ethnicity and postcode (Table 1). The average age of patients who gave consent was 62.1 years, while that of the total patient population was 65.4 years.

In addition, detailed information concerning self-reported ethnic background of KHP PCaBB participants is available (unrecorded for 35.7%). Currently, the database comprises of 44.8% White males, 15.8% Black, 2% Asian, 0.9% Mixed, and 0.7% Other ethnic background, making this collection highly ethnically diverse in comparison with other collections in UK. For instance, the UK biobank reported in July 2014 that 96.3% of their participants are of a White ethnic background [6].

Using the English Indices of Multiple Deprivation 2010 from the Office of National Statistics [7], postcodes are used to determine patient socioeconomic status (SES) based on indexes of Income, Employment and Health Deprivation, Disability, Education Skills and Training, Housing and Services, Crime, Living Environment and Total Population. Patients are then categorised into low, medium or high SES.

### Prostate cancer characteristics

Prostate cancer characteristics include Prostate Specific Antigen (PSA) levels at diagnosis, Gleason Score and TNM stage (Table 2). Gleason scores are determined either on biopsy (TRUS, TP or TURP) or on the surgical specimen. If assessed on both samples, the score from the surgical specimen is registered [8]. Patient staging can be done clinically and/or pathologically. However, if both are performed, pathological examination is used to determine the patient’s final staging based on the TNM criteria [9].

After assessing Gleason scores, Gleason Grade Groups were created [10]. These grades are coded into five groups of increasing prostate cancer severity: Grade 1 (13.5%), Grade 2 (45.0%), Grade 3 (20.2%), Grade 4 (8.0%) and Grade 5 (10.2%).

Using the above prostate cancer characteristics, patients are grouped into the following risk categories: Localised (low, intermediate and high risk), Regionally metastatic/Locally advanced and Distant metastases (Table 4) [11].

**Table 4:** Risk categories according to the National Comprehensive Cancer Network Practice Guidelines in 2010 [11]

Risk Categories	Definition
Localised prostate cancer	
Low risk	T1–2, Gleason score 2–6 and PSA <10 ng/ml
Intermediate risk	T1–2, Gleason score 7 and/or PSA 10 to <20 ng/ml
High risk	T3 and/or Gleason score 8–10 and/or PSA 20 to <50 ng/ml
Regionally metastatic/Locally advanced	T4 and/or N1 and/or PSA 50 to <100 ng/ml in the absence of distant metastases (M0 or Mx)
Distant metastases	M1 and/or PSA ≥100 ng/ml

### Treatment data

Clinical patient information includes data on treatment type, comorbidities, medications and disease outcome (Table 2). Different treatment options are listed as: surgery (65%), active surveillance (13.5%), pharmacological (Hormone therapy, 17.9%, or Cytotoxic chemotherapy, 0.4%), brachytherapy (3.3%) and specialist palliative care (0%). More than one treatment option is often used for each patient, in which case the main curative treatment is registered.

Patient comorbidities are grouped as previous/other cancers (3.6% of all participants), cardiovascular disease (39.7%), HIV or infectious disease (0.9%), hyperlipidaemia and hypercholesterolemia (15.9%), diabetes (12.6%) or psychological complaints (5.8%); with 76.3% of biobank participants having one or more comorbidities.

The average number of different medications per patient was also registered (72.2% having one or more prescribed drugs), with special attention to the drugs prescribed for erectile dysfunction (39.4% of participants).

Disease outcomes are continuously updated and are categorised into: stable disease (80.9%), discharged to GP (10.2%), treatment discharged to GP (3.1%), discharged to different hospital (0.7%), progression (1.3%), progression to metastasis (0.5%), recurrence (0.4%), death (1.1%), awaiting treatment (0.4%), refused further treatment (0.2%), treated privately (0.2%) and lost to follow-up (1.1%).

### Recruitment to clinical trials

At KHP, several prostate cancer clinical trials are conducted at any given time. Patients who meet the criteria of an active study are approached regarding possible participation in the clinical trial at the same time that they are given information about the Biobanking. Of the 549 biobank participants, 34 patients (6.2%) are also involved in a clinical trial at the time of the audit.

## Utility and discussion

The KHP PCaBB is both a clinically and demographically representative database – making it an ideal platform for research in the field of prostate cancer. The multi-ethnic foundation of our patient population grants our biobank the potential to contribute to investigations on aetiological and pathophysiological differences in prostate cancer between various ethnic groups, especially amongst UK residents of Black descent [12]. Furthermore, the clinical diversity of our repository makes it possible to explore potential variations in treatment response as well as disease markers in the different risk categories for prostate cancer.

The following are examples to highlight the possible areas where KHP PCaBB can contribute to prostate cancer research:

- **Example Study 1: Duffy antigen receptor for chemokines in black men with prostate cancer**

Afro-Caribbean men are three times more likely to develop prostate cancer than white men [13] and, are more likely to present with aggressive, higher-stage disease [14]. There are several hypotheses that surround this observation, one of which attributes this difference to reduced expression of Duffy antigen/receptor for chemokines (DARC) on the red blood cells of Black men. DARC is commonly expressed in Caucasians while approximately 95% of endemic African populations lack its expression [15].

DARC acts as a malaria parasite receptor. As such, in many African countries where malaria is fairly common, the lack of DARC expression in these populations may have resulted from an evolutionary selection process that favoured resistance towards malarial infection [15]. However, the DARC protein does confer beneficial effects – most notably, by sequestering key angiogenic chemokines including IL-8, Gro- alpha 1, MCP-1 and RANTEs from blood, thereby preventing prostate tumour development and progression [15]. Theoretically reduced expression of DARC may result in increased risk of prostate cancer incidence and severity.

In an ongoing study by Galustian and colleagues, blood samples from 100 white men and 60 black men with a diagnosis of prostate cancer were supplied by the KHP PCaBB to investigate genotypic characteristics of DARC status as well as the corresponding levels of chemokines to identify its association to cancer severity (Galustian et al., unpublished).

- **Example Study 2: Immune system involvement in Prostate Cancer pathophysiology**

A renewed interest in cancer immunotherapy over the past years has resulted in significant breakthroughs for patients with various tumour types, resulting in new systemic cancer treatments translating into true survival benefits. Although immunotherapy is currently not considered a standard treatment for advanced prostate cancer, there is a substantial body of evidence suggesting that prostate cancer is immunogenic. The Hayday laboratory at King’s College London recently showed that the body harbours a natural mechanism, termed Lymphoid Stress-Surveillance (LSS), which displays a high level of inter-individual variation and could underpin heterogeneity in immune-mediate tumour surveillance [16]. There is evidence to suggest that this rapid LSS mechanism plays an important role in prostate cancer development and progression [17, 18]. However, the status of LSS in human prostate cancer remains largely unexplored.

By using blood samples in a longitudinal study, the Hayday group is examining peripheral immune responses of prostate cancer patients undergoing treatment at KHP [19]. LSS profiles of various prostate cancer patients are being investigated including the following cohorts: ‘High Risk’ (*n* = 25) and ‘Low Risk’ (n = 25) localised prostate cancer, ‘Post Prostatectomy’ (n = 25) and patients with advanced prostate cancer receiving abiraterone acetate (*n* = 15) and docetaxel (n = 15). Patterns of LSS are currently being investigated to predict individual PCa-specific mortality and responses to treatments.

### Strengths and weaknesses

Data collected for the KHP PCaBB constructs a comprehensive demographic and clinical profile of each prostate cancer patient who has given consent for biobanking. The KHP PCaBB has thereby amassed a rich data and tissue repository that is largely reflective of both the demographic and clinical diversity within the total prostate cancer patient population seen in GSTT. This is particularly the case with respect to age, ethnicity, SES, risk category, comorbidities and treatment outcomes.

However, clinically, our biobank was not entirely reflective of the various prostate cancer treatment types as surgical patients represented more than half of biobank participants. This is most likely due to our current clinical practice in which these patients are offered an education seminar prior to their surgery. This seminar serves as an avenue through which patients receive more information regarding their surgery as well as biobanking, while having their concerns immediately addressed. We are currently developing this protocol further so a similar platform is made accessible to all other prostate cancer patients at GSTT. As a result, we believe that the clinical discrepancy between our biobank cohort and the total patient population is due to the early stage of our biobank – representativeness will improve over time with increasing numbers. Since the conduct of this audit, the number of patients has increased from 549 to 1595 in April 2017.

### Future developments

In the future, we hope to expand our repository capabilities to collect saliva and urine samples – while improving donor recruitment at all stages of the patient pathway, as mentioned above.

### Availability and requirements

Researchers who would like access to the clinical data and/or samples from KHP PCaBB will need to submit a request to the KHP Biobank Access Committee. More details can be found on www.kcl.ac.uk/prostatecancer.

## Conclusion

Data collected for the KHP PCaBB constructs a comprehensive demographic and clinical profile of each prostate cancer patient who has given consent for biobanking. The KHP PCaBB has thereby amassed a rich data and tissue repository that is largely reflective of both the demographic and clinical diversity within the total prostate cancer patient population seen at KHP, however this is not reflected by the various prostate cancer treatment types as surgical patients represented more than half of biobank participants.

## Abbreviations

DARC: Duffy antigen/receptor for chemokines
FFPE: Formalin Fixed Paraffin Embedded
GSTT: Guy’s and St Thomas’ NHS Foundation Trust
KHP PCaBB: KHP Prostate Cancer Biobank
KHP: King’s Health Partners
LSS: Lymphoid Stress-Surveillance
PBMC: Peripheral Blood Mononuclear Cells
PSA: Prostate Specific Antigen
SES: Socioeconomic Status
TP: Transperineal
TRUS: Trans-Rectal Ultrasound
TURP: Transurethral Resection Of Prostate
